# Mechanism of Centrosomal Protein 55 (CEP55) Loading Into Exosomes

**DOI:** 10.1002/jev2.70046

**Published:** 2025-02-20

**Authors:** Christian Dahlstroem, Johanna Barezani, Jing Li, Kostiantyn Sopelniak, Stefanie Muhs, Carola Schneider, Roland Thünauer, Rudolph Reimer, Sabine Windhorst

**Affiliations:** ^1^ Department of Biochemistry and Signal Transduction University Medical Center Hamburg‐Eppendorf Hamburg Germany; ^2^ Technology Platform Microscopy and Image Analysis (TPMIA) Leibniz Institute of Virology Hamburg Germany; ^3^ Center for Structural Systems Biology (CSSB) Hamburg Germany; ^4^ Technology Platform Light Microscopy (TPLM) Universität Hamburg Hamburg Germany

## Abstract

Up‐regulation of Centrosomal Protein 55 (CEP55) in cancer cells increases malignancy, and the protein can be transferred via exosomes. However, the mechanism of how CEP55 is delivered to exosomes is unknown. In this study, we addressed this issue and analysed trafficking of EGFP‐CEP55 from early to late endosomes by using high‐resolution microscopy. Our data show that endogenous as well as EGFP‐CEP55 appeared as dot‐like structures in cancer cells. However, we did not find an internalization of CEP55 into early Rab5‐ and late Rab7‐positive endosomes but only into secretory late CD63‐positive endosomes. In addition, an association of the CEP55 dots with the endoplasmic reticulum and with ALG‐2‐interacting protein X (Alix) dots was detected. Moreover, mutation of the CEP55‐Alix interaction site strongly reduced the formation of CEP55 dots as well as CEP55 localization in extracellular vesicles. In summary, our data indicate that delivery of CEP55 into exosomes does not occur by the canonical early‐to‐late endosome pathway but by Alix‐mediated recruitment to secretory late secretory CD63 endosomes.

## Graphical abstract

The cancer malignancy promoting protein CEP55 is delivered by exosomes to cancer cells. Our data show that CEP55 loading into exosomes of cancer cells is controlled by an unconventional Alix‐depending pathway that involves the ER and CD63 late endosomes. Moreover, we got indications that incorporation of CEP55‐Alix complexes into late endosomes occurs by liquid droplets.

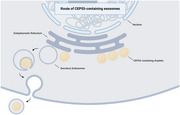

## 1 Introduction

Centrosomal Protein 55 (CEP55) is a cancer‐testis antigen whose expression is up‐regulated in a high number of cancer types. The protein stimulates PI3‐Akt signalling and promotes cellular abscission as well as chromosomal instability (reviewed in Jeffery et al. 2016). These cancer‐promoting activities are mediated by the scaffold or the microtubule (MT)‐binding activity of CEP55 (Jeffery et al. 2016; Muhs et al. 2024). Interestingly, it has been shown that CEP55 can be transferred via exosomes, thereby increasing the malignancy of lung cancer cells. Also, CEP55 is suggested as a marker for cancer‐derived exosomes (Qadir et al. 2018; Liu et al. 2023). However, the mechanism of how CEP55 is delivered into exosomes is unknown.

In inter‐phase cells, CEP55 is mainly located in the centrosome. During mitosis, phosphorylation of CEP55 results in translocation from the centrosome to the spindle MT (Muhs et al. 2024), and thereafter to the midbody, where it recruits ALG‐2‐interacting protein X (Alix) (Jeffery et al. 2016). After being located in the midbody, Alix recruits and nucleates the Sorting Complexes Required for Transport III (ESCRT) protein CHMP4b. This ESCRT III protein polymerizes into a spiral at membrane buds, initiates constriction and finally recruits the MT‐severing protein spastin, resulting in cellular abscission (Neto and Gould 2011).

In addition to the midbody, Alix is also involved in exosome biogenesis. According to the canonical model, exosomes arise from the inward budding of early endosomes (Teng and Fussenegger 2020), and since the budded vesicles remain inside the endosomes, they were named intra‐luminal vesicles (ILVs). During ILV formation, cytosolic proteins, nucleic acids and metabolites can be internalized, and are trapped inside the ILVs. For ILV formation, the early endosome membrane is invaginated and constricted with the help of the ESCRT machinery. After ILVs are formed, the endosomes are designated as multi‐vesicular bodies (MVBs). MBVs can mature to or fuse with lysosomes, and the molecules inside the ILVs are degraded, or they are transported to and fuse with the plasma membrane. Here, the ILVs are released as exosomes into the extracellular space and are internalized by acceptor cells (Gurung et al. 2021).

In addition to this canonical pathway, there are alternative pathways of exosome biogenesis, including the Alix–tetraspanin‐dependent pathway (Andreu and Yanez‐Mo 2014). Alix can specifically bind to lysophosphatidic acid (LBPA), an unconventional phospholipid of late endosomes, and based on its scaffold activity recruits the tetraspanin CD63 and also CHMP4b to late endosomes (Katoh et al. 2003; Larios et al. 2020; McCullough et al. 2008; Tang et al. 2015). The tetraspanins represent a large family of proteins that span the membrane four times. These proteins cluster at endosomal membranes and can induce membrane curvature as well as recruit cargo proteins (Andreu and Yanez‐Mo 2014).

In contrast to Alix, it is not known if also CEP55 is involved in exosome biogenesis. CEP55 has a defined Alix‐interaction site at Y187, and inside the Alix protein, the CEP55 interaction site is located in the C‐terminal proline‐rich domain. Thus, it was tempting to speculate that Alix recruits CEP55 to endosomes and also controls its incorporation in a CHPMP4‐dependent manner.

In order to analyse this hypothesis, we assessed the localization of CEP55 at endosomes by high‐resolution microscopy and indeed found a co‐localization between Alix and CEP55 at late endosomes. Moreover, our data show that the association of CEP55 with late endosomes was dependent on Alix, but we did not detect a co‐localization between CEP55 and Ra5/Rab7‐positive endosomes. Thus, delivery of CEP55 into exosomes seems to be mediated by the non‐canonical Alix–tetraspanin‐dependent pathway.

## 2 Methods

### 2.1 Cell Culture and Stable Lentiviral Knock‐Down of CEP55

OVCAR‐8 cells were cultivated in RPMI medium (72400, Gibco) containing 10% (v/v) FCS and Pen/Strep (100‐units/mL penicillin, 100‐µg/mL streptomycin). MDA‐MB‐231 and H1299 cells were cultivated in DMEM medium (41965039, Gibco) containing 10% (v/v) FCS and Pen/Strep. All cell lines were purchased from the American Type Culture Collection (ATCC).

For stable knock‐down of CEP55 in OVCAR cells, vectors from the mission shRNA system from Sigma‐Aldrich were used for lentiviral transduction. For producing lentiviral particles, 7 × 10^5^ HEK‐T‐293 were seeded into a 10‐cm Petri dish. On the following day, 1000 ng of shRNA vector, 750 ng of psPAX2, 250 ng of pMD2.G (both Addgene, Cambridge, Massachusetts) and 6 µL of FuGENE (Lot # 93576920; Roche) were mixed in 200 µL of serum‐free medium and added directly to the culture medium of the seeded cells. After 24, 48 and 72 h, the supernatant containing viral particles was collected, filtered and stored at −80°C until use. For transfection, the 48‐h supernatant was mixed with 8‐µg/mL Polybrene (Sigma) and added to the OVCAR‐8 target cells, which had been seeded at 1 × 10^5^ cells/well in a six‐well plate the previous day. Five different CEP55 shRNA constructs (TRC2‐pLKO.1 lentiviral vectors) were tested. After selection with puromycin (3 µg/mL), the two cell lines with the strongest knock‐down were selected (CEP55 sh1 and sh2). The targeted sequence of sh1 (TRCN000006197) is CCGGGCAGGCATGTACTTTAGACTTCTCGAGAAGTCTAAAGTACATGCCTGCTTTTTG. Sh2 (TRCN0000061975) targets the sequence CCGGGCAGCATCAATTGCTTGTAATCTCGAGATTACAAGCAATTGATGCTGCTTTTTG. Cells stably expressing scrambled shRNA were used as controls. Knock‐down was verified by Western blotting.

#### 2.1.1 Trafficking and Speed Analysis of CEP55 Dots

For the trafficking of CEP55 dot‐like structures, time‐lapse movies were acquired over 3‐min intervals, capturing 720 frames at 250 ms. Speed analysis of CEP55 dots was performed by using the open‐source Fiji plugin TrackMate (Ershov et al. 2022). Both pixel width and height were set to 65 nm, according to the image metadata. LAP tracking parameters were defined with a maximum linking distance of 12 pixels, a gap‐closing distance of 23 pixels and a frame gap allowance of eight frames. Only tracks with more than 20 frames were used for subsequent calculations.

### 2.2 Stable Lentiviral Re‐Expression of CEP55^WT^ and CEP55^Y187A^ Into CEP55 Knock‐Down Cells

For stable re‐expression of CEP55^WT^ and CEP55^Y187A^, the genes were cloned into the LeGo‐iB_2_ Neo+ vector (LeGo), and the empty vector was used as control (a friendly gift from Kristoffer Riecken, UKE). Stable lentiviral re‐expression of the proteins into CEP55 sh1 cells was performed as follows. A total of 1000 ng of LeGo‐iB2 Neo+ vector was mixed with 750 ng of pMDLg/pRRE packaging plasmid, 250 ng of pRSVrev packaging plasmid and 2 µg of VSV‐G pCMV vector in 750 µL of serum‐free medium, followed by a 5‐min incubation at room temperature. Simultaneously, 40 µL of Lipofectamine 2000 was mixed with 750 µL of serum‐free medium and incubated for 5 min. The two mixtures were then combined and incubated for 20 min at room temperature. The resulting solution was applied to HEK‐293‐T cells seeded into 10‐cm Petri dishes, following the same protocol as described for the lentiviral knock‐down. After 24, 48 and 72 h, the virus‐containing medium was collected, filtered and transferred to OVCAR‐8 target cells. Seventy‐two hours after the last virus infection, the cells were selected using 700‐µg/mL G418. Re‐expression was analysed by Western blotting.

### 2.3 Western Blotting

Western blot analysis was performed by a standard procedure using nitrocellulose membranes. The following antibodies were used: CEP55 (anti‐rabbit, #81693, Cell Signaling), Hsc70 (anti‐mouse, sc‐7298, Santa Cruz), Alix (anti‐mouse #2171 Cell Signaling) and Flottilin (# 610820, BD).

The membranes were blocked for 30‐min RT with 5% milk powder in Tris‐buffered saline and Tween20 (TBS‐T), the primary antibodies were diluted 1:1000 in 2.5% milk powder, and incubated overnight at 4°C. The secondary antibodies against mouse (ab205719, Abcam) or rabbit (ab205718, Abcam) were diluted 1:10,000 in TBS‐T and incubated for 1 h at RT. For signal production and detection, chemiluminescence reagent (Amersham ECL Prime Western Blotting Detection Reagent, GE Healthcare Bio‐Sciences) and the Intas ECL CHEMOCAM imager were used. Band intensities were quantified using Fiji (NIH National Institutes of Health) and normalized to HSC70 or Flottilin.

### 2.4 Transient Protein Expression in Cancer Cells

2 × 10^4^ cells were seeded into chamber slides (Ibidi), and after 16 h of incubation, the cells were transfected with EGFP‐CEP55, mRFP‐Alix, mRFP‐Rab5, mRFP‐Rab7, mCherry‐CD63 or mCherry‐CHMP4b. The Rab‐constructs were a kind gift from Stefan Lindner, and mCherry‐CD63 as well as mCherry‐CHMP4b were purchased from Addgene. Genes of CEP55 and ALIX were cloned into eGFP‐N1 and pDsRed‐N1, respectively, using SLIC (Jeong et al. 2012). Point mutants CEP55^Y187A^ and ALIX^Y806A^ were introduced using the Q5 Site‐Directed Mutagenesis Kit (NEB). Final plasmids were sequenced by Microsynth Seqlab.

For transient transfection, the K2 Transfection Reagent from Biontech was used. Prior to transfection, 2.5 µL of K2‐Multiplier were added directly to the culture medium (200 microliter) and incubated for 2 h in a CO_2_ incubator. Following incubation, 250 ng of plasmid DNA was mixed with 1.2 µL of K2 Transfection Reagent in 15 µL of serum‐free medium, incubated for 20 min at RT and then added to the culture medium of the seeded cells. After 24 h of incubation, living cells were imaged or fixed with 4% paraformaldehyde/4% sucrose.

### 2.5 Fluorescence Microscopy

SoRa spinning disc microscopy of cells growing in eight‐well Ibidi glass bottom μ‐slides was carried out using a Nikon Ti2 microscope equipped with a Yokogawa CSU W1 spinning disc unit including a SoRa optical pixel re‐assignment unit, a 100x NA 1.49 oil immersion objective and Hamamatsu Orca Fusion cameras.

For confocal microscopy and correlative light and electron microscopy (CLEM), cells were grown in plastic‐bottomed dishes (Ibidi μ‐Dish 35 mm, high Grid‐500), fixed with 2% formaldehyde and imaged without the lid to enable DIC. Imaging was performed on a Nikon A1+ microscope using a 40×/0.95 air objective. For high‐resolution light microscopy, the Nikon AX/AX R, Nikon Spatial Array Confocal (NSPARC) system with a Plan Apo IR 60x WI DIC N2 silicone objective and AX camera were used. Additionally, the light microscopy data were denoised and deconvolved using the Nikon NIS‐Elements v5.42 AI plugin.

For vesicle trafficking and for widefield microscopy, the IXplore Live microscope from Olympus with 100x oil X‐Line or 20x Semi Apochromate objectives were used.

### 2.6 Electron Microscopy

Serial block‐face scanning electron microscopy (SBFSEM) was performed as described earlier (Flomm et al. 2022).

To re‐localize fluorescent signals using CLEM, lipid droplets were utilized as fiducial markers. The SBFSEM datasets were pre‐aligned using Gatan Digital Micrograph v3.2 and then cropped, registered (using the Stackreg plugin) and denoised (using the DenoiseEM plugin) in Fiji. 3‐D re‐construction was performed with the Tescan 3D Analysis Suite v1.7.2.

### 2.7 Immunofluorescence

2 × 10^4^ cells were seeded into chamber slides (Ibidi), fixed with 4% paraformaldehyde/4% sucrose for 10 min at 37°C, permeabilized with 0.3% Triton‐X‐100/PBS for 5 min at RT, washed three times with 0.03% Triton‐X‐100/PBS, blocked with 2.5% BSA for 1 h and incubated with primary antibodies diluted 1:200 (anti‐calnexin (Santa Cruz sc‐70481), anti‐TGN38 (R&D Systems, MAP49‐44), anti‐beta‐tubulin (Sigma, T4026) and anti‐CD9 (CoralLite594‐conjugatd, Proteintech, CLS94‐60232)) in PBS/0.03% Triton‐X‐100/1.25% BSA overnight at 4°C. After washing three times with 0.03% Triton‐X‐100/PBS, the samples were incubated for 1 h at RT with secondary 568‐Alexa‐fluor‐coupled antibodies diluted 1:2000 in PBS/0.03% Triton‐X‐100/1.25% BSA. Except for the CD9 antibody, which is already conjugated (see above). After washing the cells three times with 0.03% Triton‐X‐100/PBS, the samples were covered with PBS and analysed by fluorescence microscopy.

### 2.8 Isolation of Extracellular Vesicles (EV) and Nanoparticle Tracking Analysis (NTA)

5 × 10^6^ OVCAR‐8 cells were seeded to T175 flasks in an EV‐free medium, and after 4 days of incubation under standard conditions, vesicles were purified by differential centrifugation at 4°C. First at 500 g for 10 min to eliminate detached cells, following 10,000 g for 30 min to pellet cell debris, and finally at 100,000 g for 90 min to harvest EVs. The EV pellet was finally washed for cellular assays with 1× PBS pH 7.4 containing EDTA‐free Protease Inhibitor Cocktail tablets (cOmplete EASYpack, Roche #04693132001).

For nanoparticle tracking analysis (NTA), the EVs were diluted in 100‐µL PBS and thereafter diluted again 1:1000 in PBS. These samples were analysed using the Nano‐Sight LM14. Samples were measured three times, and the values were normalized to the respective cell number the EVs derived from.

### 2.9 Recombinant Expression and Purification of CEP55

CEP55 and Alix genes were codons optimized for expression in *E. coli* strains and cloned into the psf421 vector to get His‐GFP or His‐mPlum fusion proteins. Proteins were expressed in Rosetta 2(DE3)pLysS *E. coli*. The bacteria were incubated in Terrific Broth (TB) medium, shaken at 37°C until OD600 of one, and protein expression was induced with 0.1‐mM IPTG. After incubation overnight at 16°C, bacteria were harvested (3700 × *g*, 10 min, 4°C) and washed with PBS. For cell lysis, the pellet was re‐suspended in a lysis buffer (20‐mM HEPES pH 7.5, 400‐mM NaCl, 1‐mM EDTA, 1‐mM DTT, 0.5‐mM benzamidine). After disruption with the cell homogenizer (Constant Systems Ltd CF1) at 1.8 kbar, 1‐mM PMSF and 0.1% Triton‐X‐100 were added. Prior to centrifugation (48,000 × *g*, 30 min, 4°C), 25‐mM imidazole was added and incubated for 5 min. The protein lysate was incubated with equilibrated Ni‐NTA Agarose (R901, Invitrogen) for 1 h at 4°C. Beads with lysate were applied to a column and washed with 20‐mM HEPES pH 7.5, 400‐mM NaCl, 0.1% Triton‐X‐100 and 25‐mM imidazole. Followed by incubation with 10‐mM ATP and 20‐mM MgCl_2_ in 20‐mM HEPES pH 7.5, 400‐mM NaCl and 25‐mM imidazole for 25 min. Beads were washed with increasing imidazole concentrations (35‐, 50‐, 62.5‐ and 75‐mM imidazole). Elution was performed twice with 20‐mM HEPES pH 7.5, 400‐mM NaCl and 130, respectively, 150‐mM imidazole and 1‐mM PMSF. Proteins were dialysed overnight at 4°C to 20‐mM HEPES pH 7.5, 250‐mM NaCl and 1‐mM DTT. The protein concentration of elution fractions was quantified with a BSA standard by SDS‐PAGE stained with Roti Blue quick (4829.1, Roth). Proteins were stored at 4°C on ice.

## 3 Results

### 3.1 Cellular Distribution of CEP55

**FIGURE 1:**
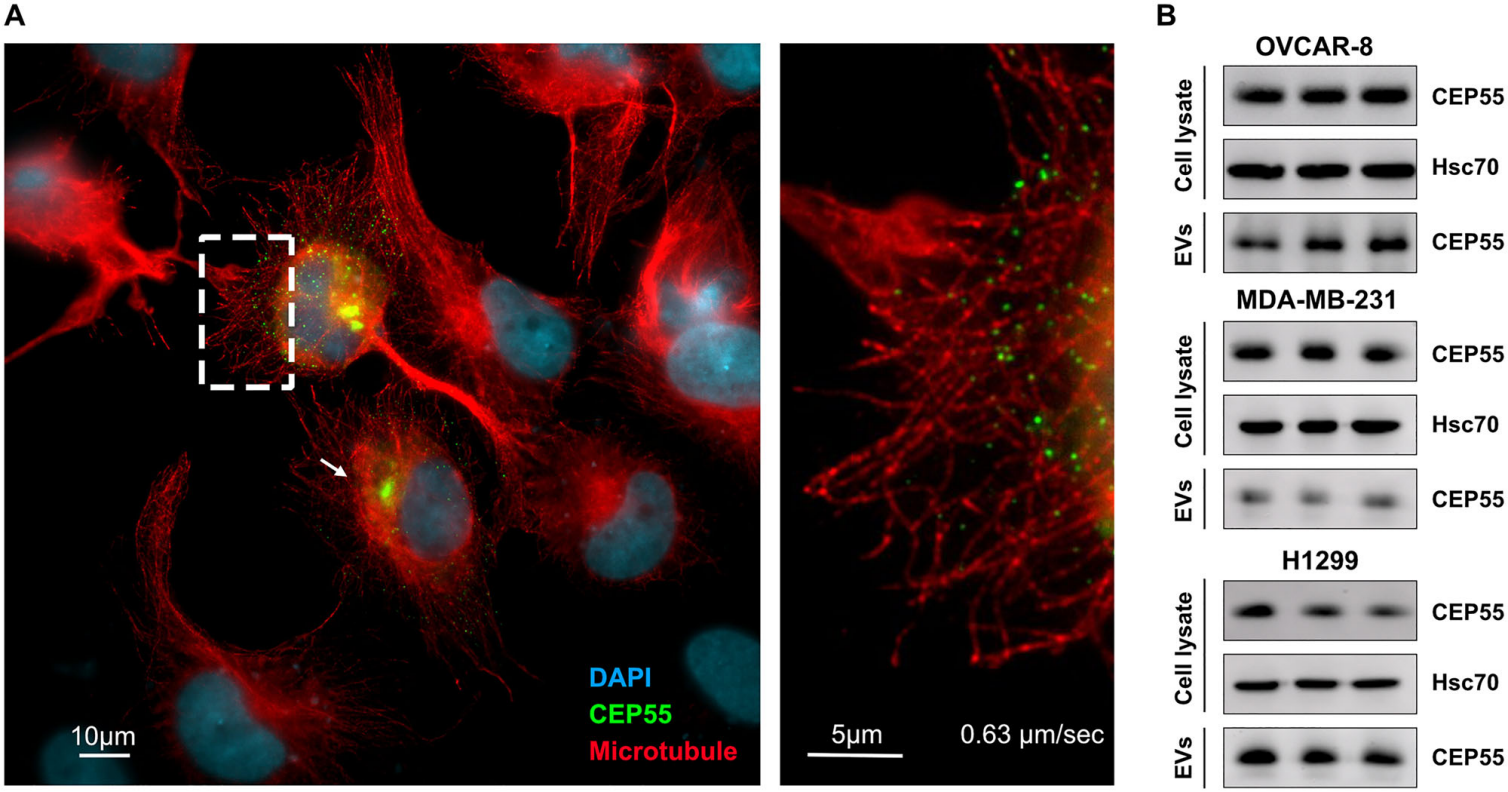
Localization of CEP55 in cancer cells. (A) OVCAR‐8 cells were transfected with EGFP‐CEP55 (green), and stained with an Alexa‐fluor568‐coupled antibody against beta‐tubulin (red) as well as with DAPI (blue). Shown is one representative fluorescence microscope image (Olympus IXplore Live‐System). (B) Cell lysates and EV fractions from different cancer cell lines were prepared, and the CEP55 levels were analysed by Western blotting, Hsc70 served as a loading control. The samples are shown as triplicates.

In order to analyse a potential CEP55 localization to cellular endosomes, EGFP‐CEP55 was expressed in ovarian cancer OVCAR‐8 cells, and CEP55 localization was assessed by fluorescence microscopy. At short exposure times, CEP55 is only detectable in the centrosomes of interphase cells (see cell marked with a white arrow in Figure 1A). However, at longer exposure times, when the centrosomal CEP55‐signal is already overexposed, EGFP‐CEP55 shows a dotted localization pattern (see enhancement in Figure 1A), which was also found in lung cancer H1299 and in breast cancer MDA‐MB‐231 cells (Figure S1). To assess the potential endosomal characteristics of the CEP55‐containing dots, their cellular distribution and speed were analysed. Analysis of EGFP‐CEP55‐transfected, MT‐stained OVCAR‐8 cells revealed that the EGFP‐CEP55 signals showed a perinuclear clustering and were also located at the cell periphery in close proximity to MTs (Figure 1A). Moreover, life‐cell imaging showed that the CEP55 dots moved with an average speed of 0.63 µm/s, which is in a similar range as the speed of early and late endosomes (Toshima et al. 2006).

In addition, we validated endogenous dot‐like CEP55 localization (Figure S2) and its localization in extracellular vesicles (EVs) (Figure 1B).

In summary, in addition to the centrosome, CEP55 also shows a dot‐like localization in cancer cells.

### 3.2 Localization of CEP55 to Endosomes

**FIGURE 2:**
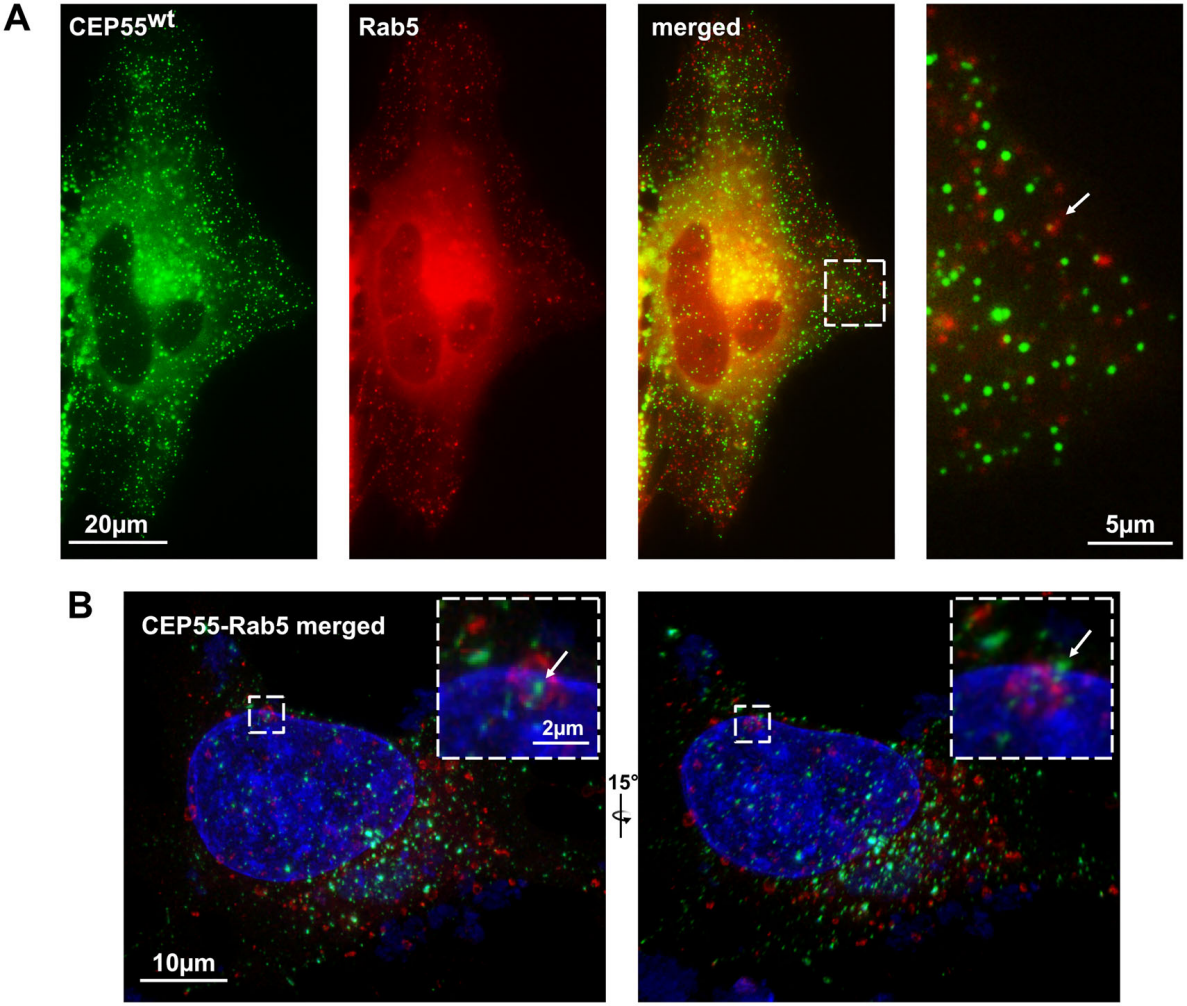
CEP55 does not co‐locate with early endosomes. (A) OVCAR‐8 cells were co‐transfected with EGFP‐CEP55 (green) and mRFP‐Rab5 (marker for early endosomes, red), fixed and analysed by fluorescence microscopy (Olympus IXplore Live‐System). (B) The same as in (A) but here the cells were imaged by high‐resolution microcopy using Nikon AX/AX R with Nikon Spatial Array Confocal (NSPARC) system. Also, Z‐stacks were performed and 3‐D‐re‐constructions were created. The right panel shows a 15% rotation into the *Z* position. The Rab5 endosome with the most adjacent CEP55 dot is shown in magnification and marked by an arrow.

In case the CEP55‐containing dots are later secreted as exosomes, we expect a co‐localization between these dots and endosomes. To assess this, cells were co‐transfected with EGFP‐CEP55 and a marker protein for early endosomes (mRFP‐Rab5). However, in fixed cells, we only found a low and not very clear co‐localization of CEP55 with Rab5 (see arrow in magnification of Figure 2A). This result led us to analyse EGFP‐CEP55 and mRFP‐Rab5 co‐transfected cells in more detail by high‐resolution microscopy and by re‐construction of 3‐D images from Z‐stacks. However, although it seemed that CEP55 had been internalized into early endosomes (Figure 2B, left panel, arrow), in a slightly different position of the image, the apparent co‐localization between CEP55 and Rab5 disappeared (Figure 2B, right panel, arrow). This was true for all images we analysed, thus it seems that CEP55 dots are not incorporated into early endosomes.

**FIGURE 3:**
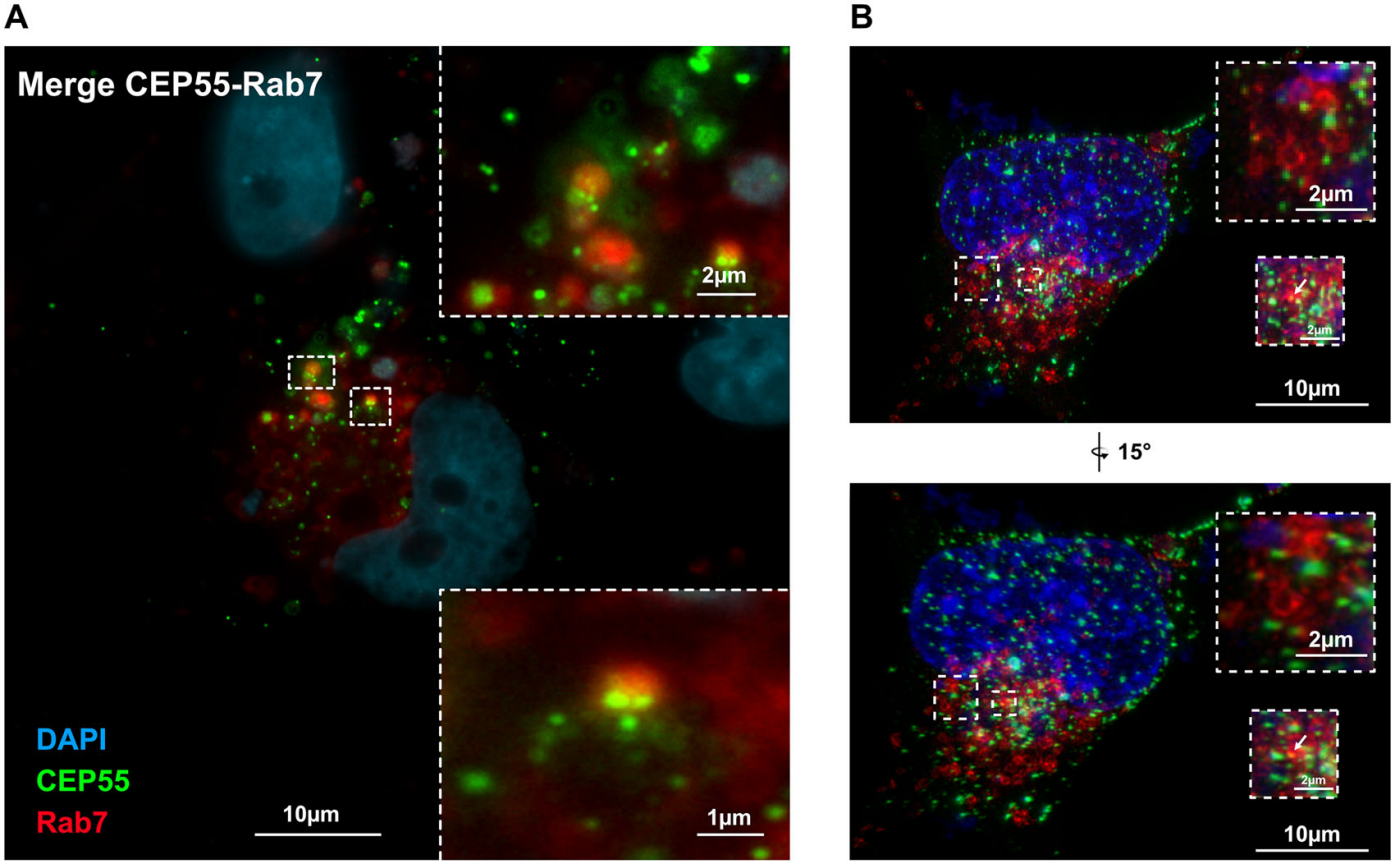
CEP55 localization at late Rab7‐positive endosomes. (A) OVCAR‐8 cells were co‐transfected with EGFP‐CEP55 (green) and mRFP‐Rab7 (marker for late endosomes, red), fixed and analysed by fluorescence microscopy (Olympus IXplore Live‐System). (B) The same as in (A) but here the cells were imaged by high‐resolution microcopy using Nikon AX/AX R with Nikon Spatial Array Confocal (NSPARC) system. Also, Z‐stacks were performed and 3‐D‐re‐constructions were created. The lower shows panel a 15% rotation into the *Z* position. The Rab7 endosome with the most adjacent CEP55 dot is shown in magnification in the lower panel and marked by an arrow. The upper magnification also shows Rab7 endosomes with loosely associated CEP55 dots. This image reveals that the CEP55 dots had not been incorporated into the Rab7 endosomes.

Since early endosomes mature to late endosomes/MVBs, we next analysed cells transfected with EGFP‐CEP55 and Rab7 (MVB/late endosome/lysosome marker) or CD63 (a tetraspanin, and a marker for secretory MVBs). Again, only a low co‐localization frequency was detectable between CEP55 and Rab7 in fixed cells (see magnification in Figure 3A), but in this case, analysis by high‐resolution microscopy confirmed the association between CEP55 and Rab7 (see magnification and arrow in Figure 3B). However, we did not detect any CEP55 signals inside the lumen of Rab7‐positive endosomes (see magnification, upper panel in Figure 3B).

**FIGURE 4:**
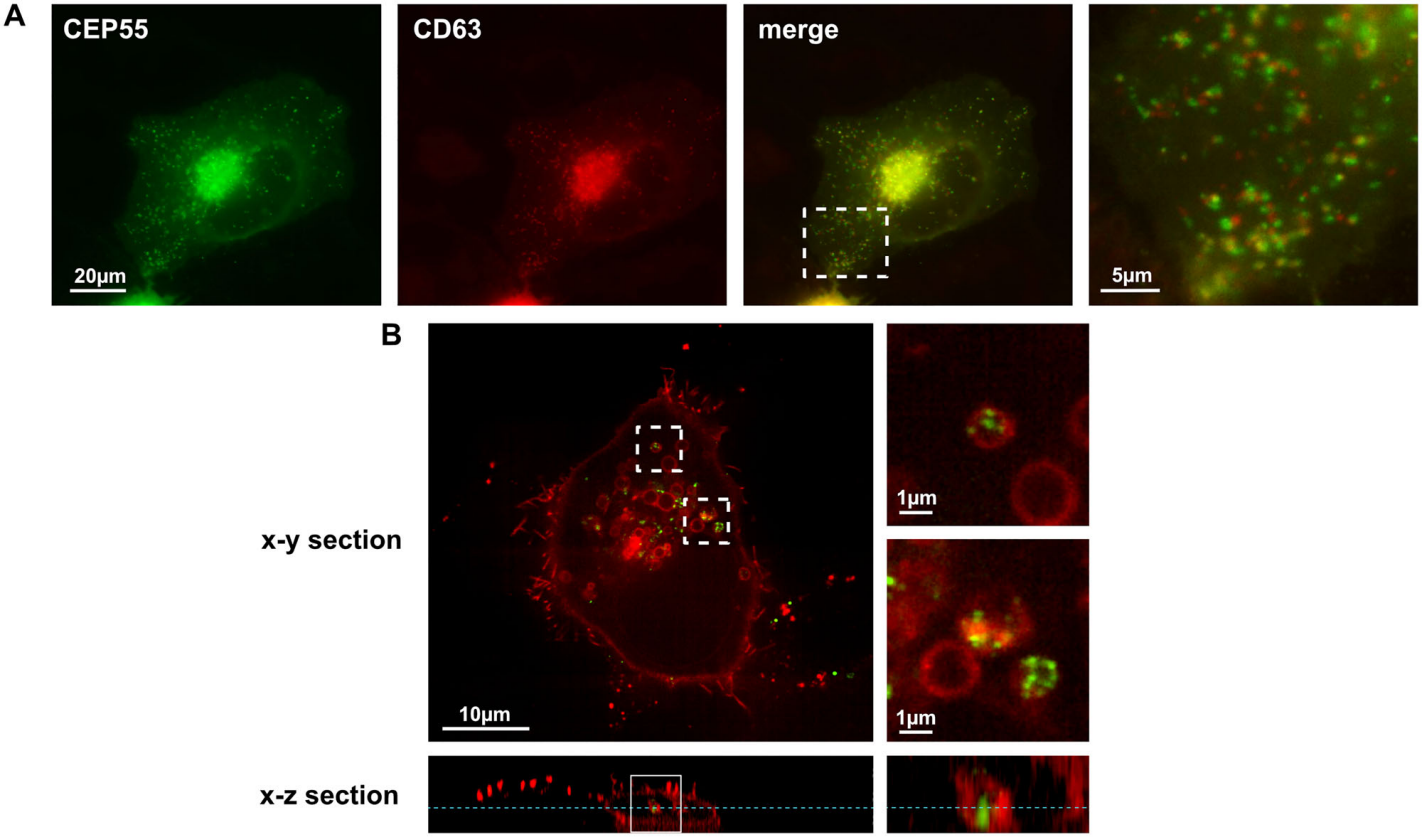
CEP55 is associated with secretory CD63‐positive MVBs. (A) OVCAR‐8 cells were co‐transfected with EGFP‐CEP55 (green) and mCherry‐CD63 (marker for secretory late endosomes, red), fixed and analysed by fluorescence microscopy (Olympus IXplore Live‐System). (B) The same as in (A) but here the cells were imaged by SoRa spinning disc microscopy (Nikon Ti2 microscope). In the right panels, exemplary enlarged images of CD63‐endosomes co‐locating with CEP55 are shown, and in the lower panel, an *x*–*z*‐position of the endosome that had incorporated a CEP55 dot.

On the other hand, most CD63‐positive vesicles were associated with CEP55 particles, although there was no complete overlay (magnification in Figure 4A). Rather, it seemed that the CEP55 and the CD63‐positive vesicles stuck to each other. This assumption was confirmed by high‐resolution SoRa microscopy (Figure 4B, magnification upper panel). However, in this case, a few CD63‐positive vesicles show CEP55 signals inside the lumen (Figure 4B, magnification lower panel, and also *x*–*z*‐section). In order to analyse whether CEP55 also co‐locates with other tetraspanins, EGFP‐CEP55 transfected cells were stained with an antibody against CD9 and analysed by fluorescence microscopy. As shown in Figure S3, no co‐localization between EGFP‐CEP55 and CD9 was visible, indicating that CEP55 does not bind to tetraspanins in general.

In summary, our data show that CEP55 did not co‐locate with early Rab5‐positive endosomes, and only a few CEP55 dots were associated with Rab7‐positive late endosomes. On the other hand, a high fraction of CEP55 dots was associated with secretory CD63‐positive MVBs, and some of them were internalized into the lumen of these endosomes.

### 3.3 CEP55 particles Are Associated With the Endoplasmic Reticulum, Are Not Enclosed by a Membrane and the Recombinant CEP55 Phase Separates

**FIGURE 5:**
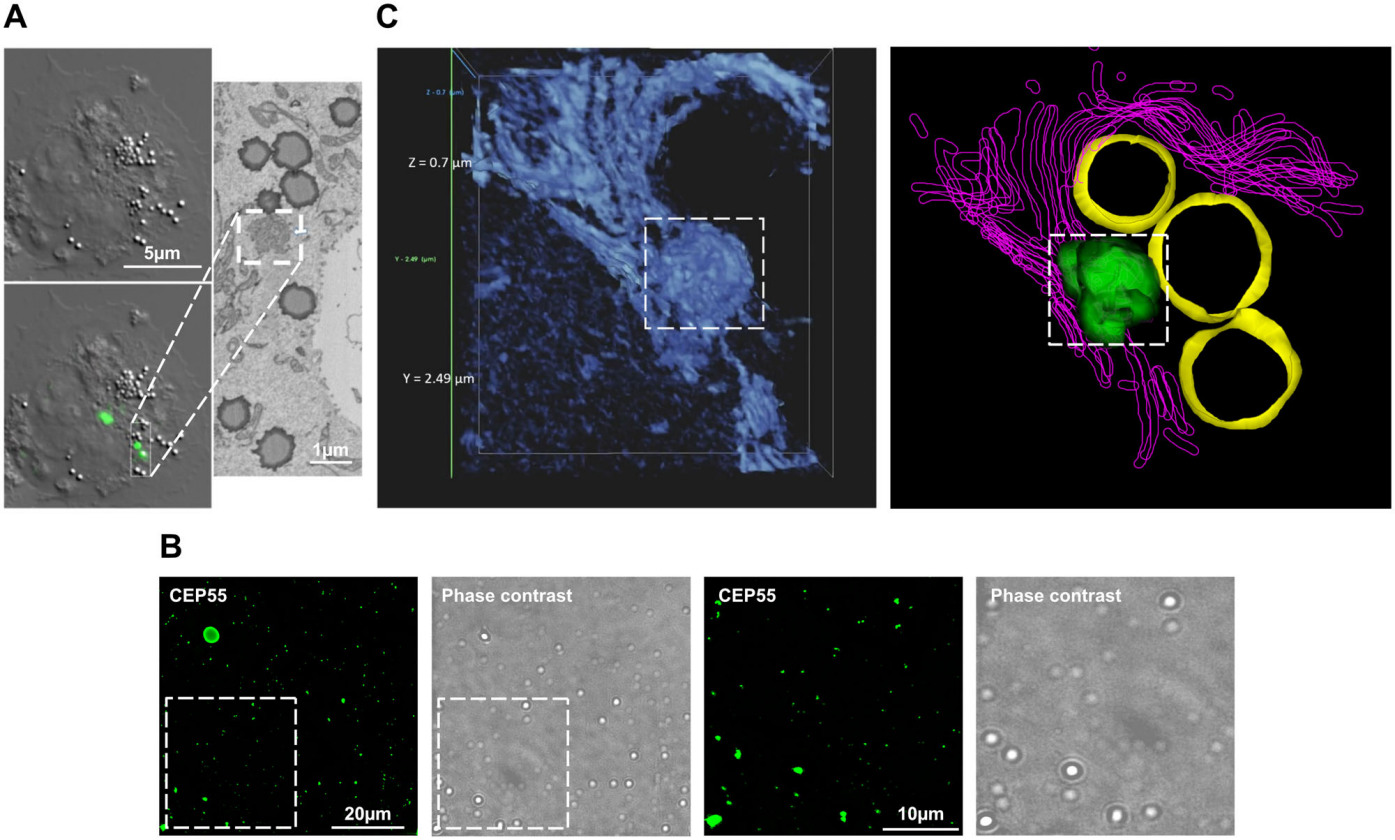
CEP55‐particles associated with the ER membrane. (A) Cells were transfected with EGFP‐CEP55 (green), and imaged by fluorescence and bright field microscopy to identify lipid droplets adjacent to CEP55 dots. Right panel shows a transmission electron microscopy (TEM) image, the lipid droplets were used to locate CEP55‐dots (marked by a white square). (B) 2‐mg/mL recombinant EGFP‐CEP55 was diluted in 5% PEG and analysed by microscopy. Left panels: EGFP‐channel, right panels: phase contrast. (C) Left panel: 3‐D re‐construction of the enlarged CEP55 dots marked by a dotted square (Flomm et al. 2022). Here, the lipid droplets are deleted to focus on ER‐like structures. In the right panel, the cellular structures were marked by colours, and the lipid droplets are included (yellow), ER‐like structures are shown in magenta, and the EGFP‐CEP55 dot in green.

To analyse the CEP55‐containing particles in more detail, EGFP‐CEP55 transfected cells were analysed by transmission electron microscopy (TEM), and the EGFP‐CEP55‐positive vesicles were identified by their position near lipid droplets. Interestingly, the EM images revealed that the CEP55 dots were not surrounded by a membrane (see dotted square in Figure 5A), indicating that CEP55 forms liquid droplets. It has been already shown that in vitro CEP55 is incorporated into Alix droplets (Elias et al. 2023), but it was not known if CEP55 phase also separates in the absence of Alix. In order to analyse this, CEP55 and Alix were recombinantly expressed in bacteria as EGFP‐ or mPlum‐His‐fusion proteins, and purified by nickel‐chelate affinity chromatography. To analyse phase separation, the same conditions as published previously for Alix were applied (Elias et al. 2023), and EGFP‐CEP55 was diluted in 5% PEG in different concentrations. Indeed, in a concentration of 2 mg/mL EGFP‐CEP55 formed liquid droplets, having a size of 1–2 µm (Figure 5B). We also reproduced the finding of Elias et al. (2023), by showing the co‐localization between EGFP‐CEP55 and mPlum‐Alix in liquid droplets (Figure S4). In salty aqueous solutions (PBS, TBS) as well after dilution of 5% PEG with PBS (1:1), EGFP‐CEP55 was diluted (buffer) or precipitated (diluted PEG) (Figure S4A,B).

To get more information about the microenvironment of the CEP55 droplets, EGFP‐CEP55‐transfected samples were sliced into different sections and analysed by TEM. Thereafter, a 3‐D re‐construction of the different TEM sections was performed. In Figure 5C's first panel, the lipid droplets were deleted to focus on an ER‐like structure, and the CEP55 droplet is marked by a dotted square. The right panel of Figure 5C includes the lipid droplets, which are marked in yellow, the ER‐like structures are shown in magenta and the CEP55 dot is in green. To analyse if the CEP55 dots are associated with the ER or with the trans‐Golgi network (TGN), EGFP‐CEP55‐transfected cells were stained with an ER‐ (Calnexin) and a TGN marker (TGN38). Analysis by fluorescence microscopy revealed a co‐localization between CEP55 and Calnexin but not with CEP55 and TGN38 (Figure S5).

Together, our results show that cellular CEP55 dots are not surrounded by a membrane, and the recombinant CEP55 phase separates in 5% PEG at a concentration of 2 mg/mL. In addition, 3‐D images of TEM stacks revealed that nuclei near CEP55 droplets were associated with the ER membrane.

### 3.4 Alix Determines the Dot‐Like Localization of CEP55

**FIGURE 6:**
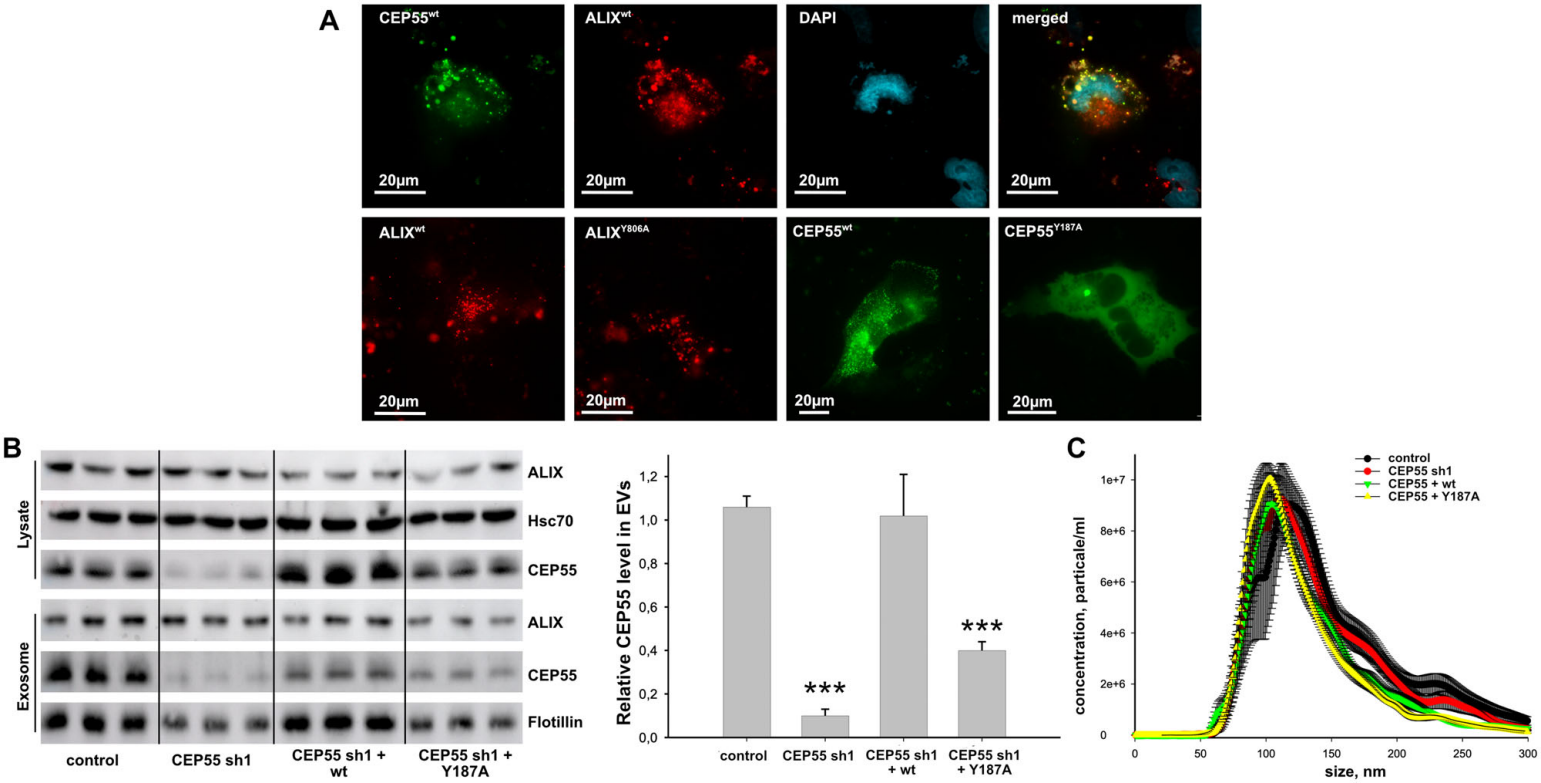
CEP55 localization depends on its interaction with Alix. (A) Cells were co‐transfected with EGFP‐CEP55 (green) and mRFP‐Alix (red) or with Alix and CEP55 mutants and analysed by fluorescence microscopy (Olympus IXplore Live‐System). (B) Cell lysates and EVs were analysed by Western blotting. For cell lysates Hsc70, and for EVs Flottilin served as loading control. Right panel shows the quantification of CEP55 signals derived from EVs normalized to Flottilin signals. (C) The concentration of EVs was analysed by NTA. Shown are the mean ± SEM of six different experiments.

Since CEP55 specifically interacts with Alix by binding to its C‐terminal proline‐rich domain (Larios et al. 2020), we next analysed a potential co‐localization between CEP55 dots and Alix. For this purpose, mRFP‐Alix and EGFP‐CEP55 were co‐expressed in H1299 cells and analysed by fluorescence microscopy. Indeed, we found that Alix was located in 52% of the CEP55 dots (Figure 6A). To validate that this co‐localization depends on the specific interaction between CEP55 and Alix, we compared CEP55 and Alix mutants defective in Alix (CEP55‐Y187A) or CEP55 binding (Alix‐Y806A). Interestingly, the Alix mutant Y806A still showed a dot‐like cellular localization but CEP55 Y187A was only located in the centrosome, and even at higher exposure times, CEP55 did not appear as dots (data not shown). Thus, Alix seems to determine the dotted CEP55 localization but not the other way around.

In order to show whether also the exosomal localization of CEP55 depends on its interaction with Alix, we analysed CEP55 localization in EVs from control OVCAR‐8 cells, from cells with stable CEP55 downregulation (CEP55 sh1), as well as from shRNA cells re‐expressing wtCEP55 (CEP55 sh1 + wt), or CEP55Y187A (CEP55 sh1 + Y187A) by Western blotting. Normalized evaluation of band intensity revealed that the level of Alix was not significantly altered (data not shown) in EVs derived from cells re‐expressing CEP55Y187A compared to those from control cells and cells re‐expressing wtCEP55. On the other hand, the level of CEP55 was significantly reduced in CEP55Y187A cells compared to the respective controls (Figure 6B). Finally, EVs from conditions media were enriched by serial ultracentrifugation, and the EV‐concentration analysed by NTA but as shown in Figure 6C, no significant differences between CEP55 control and CEP55‐manipulated cells were found. However, the isolation of EVs by ultracentrifugation does not allow to fraction EV sub‐populations (Durak‐Kozica et al. 2022), therefore, we cannot exclude that CEP55 alters the concentration of particular EV sub‐populations.

In summary, our data show that the dot‐like cellular localization of CEP55 as well as its localization in EVs depends on its interaction with Alix. However, the localization of Alix inside EVs as well as the EV concentration do not seem to be regulated by the CEP55 level.

## 4 Discussion

CEP55 is highly enriched in tumour cell–derived exosomes (Qadir et al. 2018; Liu et al. 2023), and Liu et al. (2023) revealed that invasion of lung cancer cells was promoted by CEP55‐containing exosomes. However, the mechanism of how CEP55 is internalized into endosomes/exosomes has not been investigated yet.

In this study, we addressed this issue and showed that endogenous as well as overexpressed CEP55 appeared as dots in cancer cells. Initially, we assumed that these dots represented early endosomes that had incorporated CEP55 and started to mature into late endosomes/MVB. However, high‐resolution microscopy and 3‐D re‐constructions did not show a co‐localization between CEP55 and early Rab5‐endosomes. Also, a very low co‐localization frequency between late Rab7‐positive endosomes and CEP55 dots was found in clusters around the nuclei but these endosomes had not incorporated the CEP55 dots. On the other hand, a high number of CEP55 particles were associated with CD63‐positive secretory endosomes, and we also could show internalization of CEP55 into this endosome population. These results strongly indicate that CEP55 delivery into exosomes does not occur by the canonical early‐to‐late endosomes pathway (Gurung et al. 2021).

To get deeper inside into CEP55 exosome delivery, EGFP‐CEP55 transfected cells were analysed by TEM, and in order to clearly locate EGFP‐CEP55, the dots near the lipid droplets were selected. Although we expected to find the dots embedded inside MVBs, they were membrane‐less and associated with the ER membrane. In addition to the images shown in Figure 5A–C, we analysed further cells but could not detect any CEP55 dots inside MVBs (data not shown). This finding further supports our conclusion that CEP55 is not delivered to exosomes by the canonical pathway.

Since it was recently shown that the interaction partner of CEP55, Alix phase separates and also incorporates CEP55 into these liquid droplets (Elias et al. 2023), we hypothesized that the membrane‐less CEP55‐dots may represent CEP55‐liquid droplets (Iwashita, Mimura, and Shiraki 2018; Lee and Bahmanyar 2020). Indeed, like Alix, also recombinant CEP55 phase separated in high concentrations (2 mg/mL) when diluted in 5% PEG, although these liquid droplets were smaller than Alix liquid droplets (Elias et al. 2023). However, in cells, the dot‐like CEP55 localization was completely abolished after overexpressing a CEP55 mutant with a defective Alix interaction site, indicating that CEP55 dots only are formed after interaction with Alix. Possible explanations for this finding are that after overexpression in cells, CEP55 alone does not reach the concentration sufficient to phase separate or that CEP55 only phase separates after association with membranes (Ditlev 2021). Both assumptions are possible; the interaction between CEP55 and Alix may increase the protein concentration, and Alix selectively binds to the membranes of late endosomes (Larios et al. 2020), and thereby recruits CEP55.

Alix is also required to locate the tetraspanin CD63 as well as CHMP4b to late endosomes (Larios et al. 2020), and we found a strong co‐localization between CEP55 and CD63 as well as with CEP55 and CHMP4b (Figure S6). Thus, it is very likely that Alix scaffolds CEP55, CHMP4b and CD63 at the membrane of late endosomes. Since we did not find a co‐localization between CEP55 and CD9, it seems that tetraspanins are not recruited to the CEP55‐Alix complex in general. However, the mechanism of how this protein complex is incorporated into the endosomes is unknown. Here, two different scenarios are possible as follows: (1) Internalization of the complex is driven by CD63 and CHMP4b‐mediated vesicle formation and abscission, (2) the complex is passively incorporated as liquid droplet by tension forces as recently shown by Gosh et al. for uptake of artificial nanodroplets into nanovesicles (Ghosh, Satarifard, and Lipowsky 2023). Here, the authors reveal that the incorporation of nanodroplets into nanovesicles is driven by tension forces between the droplets and the vesicles, and internalization is limited by the size of the nanodroplets. To validate these assumptions, an Alix mutant with defective CHMP4a or CD63 interaction site must be overexpressed in cells, and localization of the protein complex inside endosomes must be analyzed. Also, the speed of CEP55‐droplet incorporation as well as the droplet size could be assessed to distinguish between active (Alix‐CHMP4) and passive (tension forces) incorporation.

Another important question was how the ER membrane is involved in the delivery of CEP55 into late endosomes. Our finding that the formation of cellular CEP55 droplets was dependent on Alix strongly indicates that the CEP55 dots detected by TEM include Alix proteins as well as the Alix binding partners CD63 and CHMP4a. This also would explain why all these proteins show a perinuclear clustering. Interestingly, also late endosomes can bind to the ER by membrane contact sites, where they undergo a GTPase switch determining the fate of endosomes to the lysosomal or secretory pathway (Lee et al. 2020). Thus, it might be possible that ER‐associated CEP55/Alix‐containing droplets interact with LBPA at late endosomes, and switch the protein complex from the ER to the membrane of late endosomes. Future experiments will show if this hypothesis holds true.

In summary, our data show that CEP55 incorporation into endosomes does not occur by the canonical early‐to‐late endosome pathway. The CEP55 dots were not embedded by a membrane, accumulated at the ER membrane, and were only internalized by CD63‐postive secretory late endosomes. Since the CEP55 dot formation was dependent on Alix, and Alix also scaffolds CD63 and CHMP4b to the membrane of late endosomes, the localization of CEP55 at CD63‐positive endosomes most likely is due to the scaffolding activity of Alix. In addition, we strongly assume loading of late endosome with the CEP55/Alix/CHMP4/CD63 complex occurs at the ER membrane.

## Author Contributions

**Christian Dahlstroem**: data curation (lead), formal analysis (equal), investigation (lead), methodology(equal). **Johanna BarezaniL**: data curation (equal), investigation(equal). **Jing Li**: data curation (equal), methodology(equal). **Kostiantyn Sopelniak**: data curation (equal), formal analysis (equal), investigation (equal). **Stefanie Muhs**: data curation (equal), formal analysis (equal), investigation (equal), methodology (equal). **Carola Schneider**: methodology (equal). **Roland Thünauer**: methodology (equal). **Rudolph Reimer**: methodology (equal). **Sabine Windhorst**: Conceptualization (equal); writing–original draft (lead).

## Conflicts of Interest

The authors declare no conflicts of interest.

## Supporting information



Supporting Information

## Data Availability

The data that support the findings of this study are openly available in this manuscript.
