# Supplementary material for: Mechanism of Centrosomal Protein 55 (CEP55) Loading Into Exosomes
Source: J Extracell Vesicles. 2025 Feb 20;14(2):e70046. doi: 10.1002/jev2.70046 (PMC11840697; doi:10.1002/jev2.70046)

## Slide 1
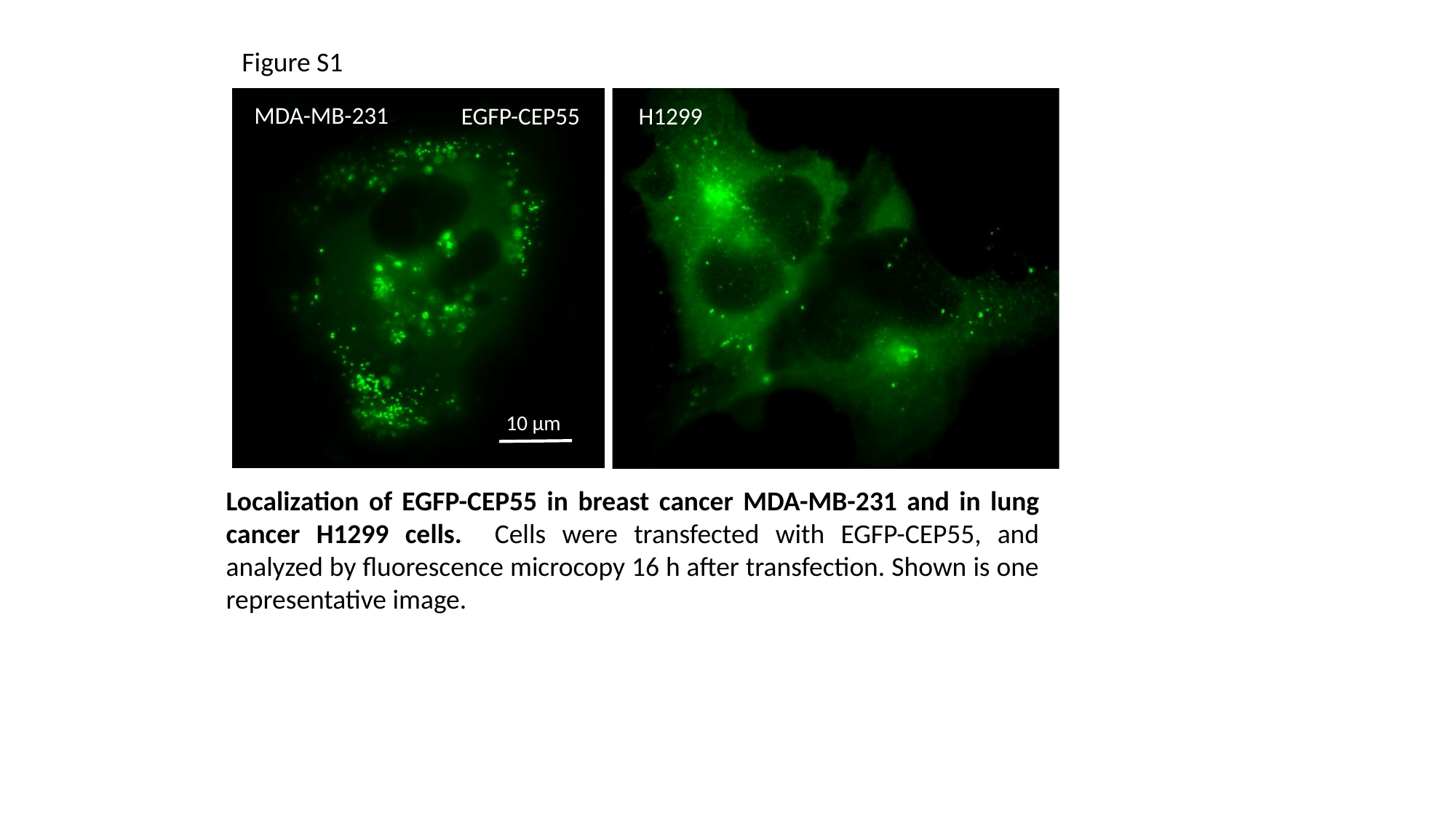

Figure S1
MDA-MB-231
EGFP-CEP55
H1299
10 µm
Localization of EGFP-CEP55 in breast cancer MDA-MB-231 and in lung cancer H1299 cells. Cells were transfected with EGFP-CEP55, and analyzed by fluorescence microcopy 16 h after transfection. Shown is one representative image.

## Slide 2
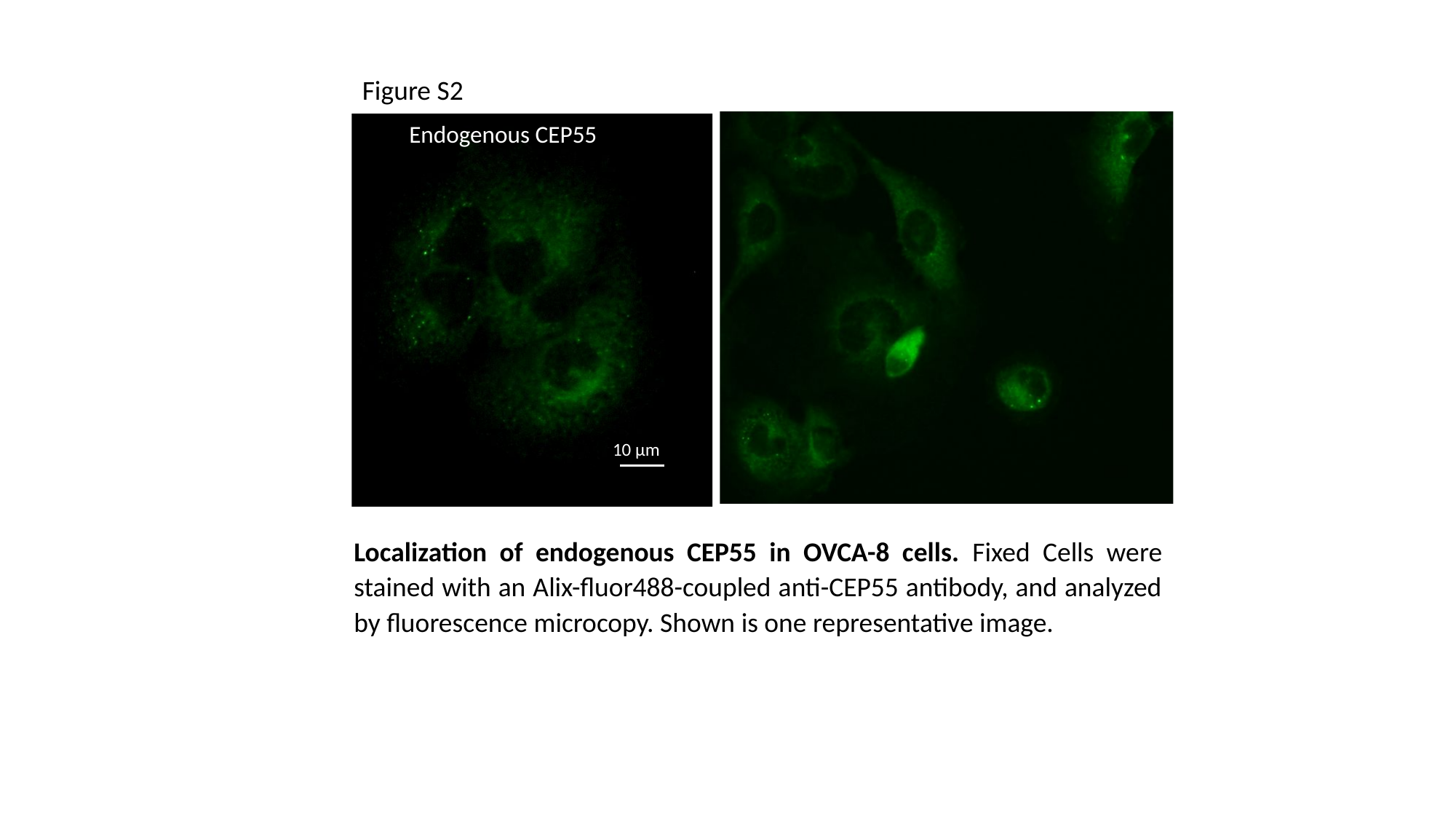

Figure S2
Endogenous CEP55
10 µm
Localization of endogenous CEP55 in OVCA-8 cells. Fixed Cells were stained with an Alix-fluor488-coupled anti-CEP55 antibody, and analyzed by fluorescence microcopy. Shown is one representative image.

## Slide 3
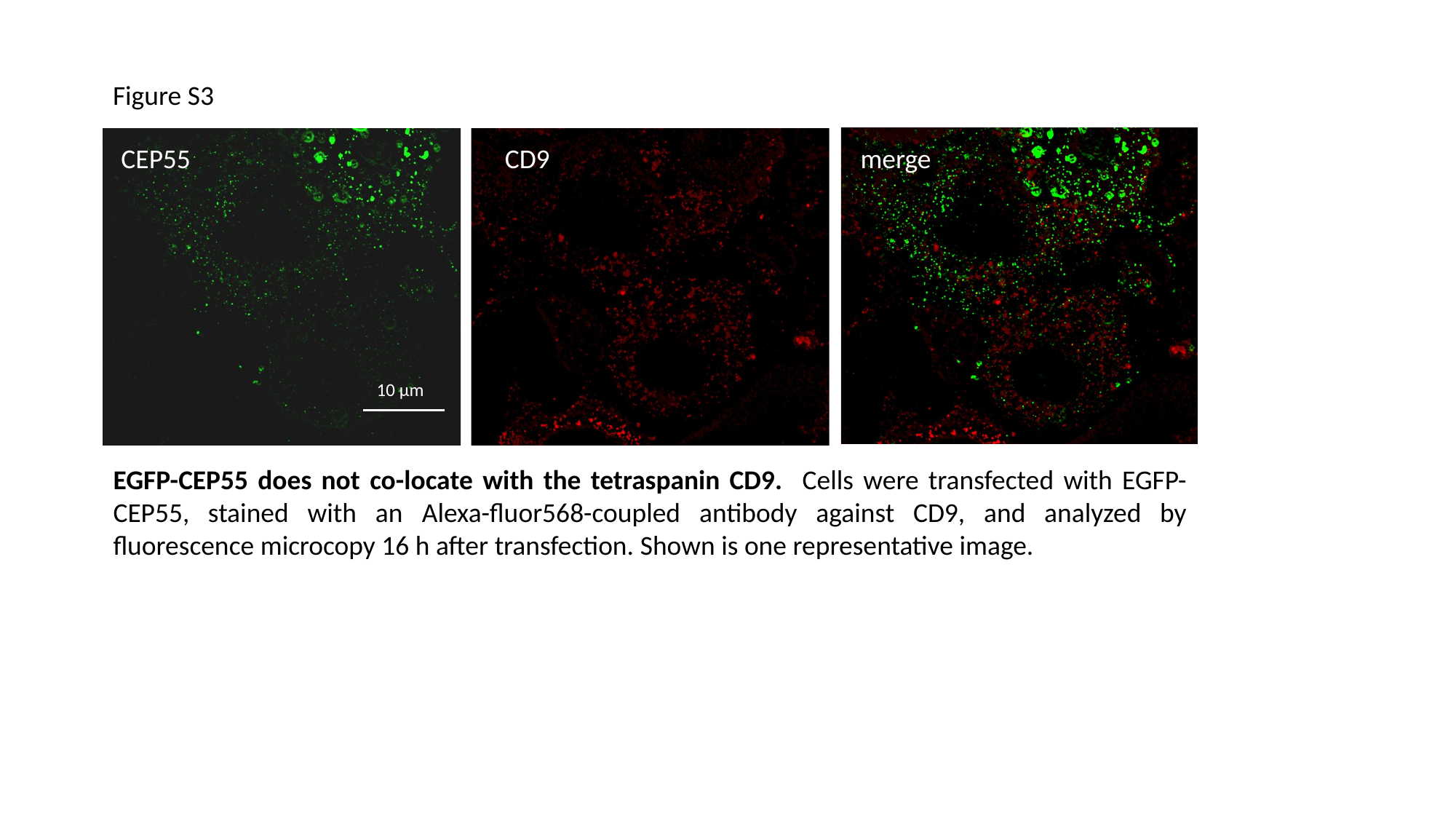

Figure S3
CEP55
CD9
merge
10 µm
EGFP-CEP55 does not co-locate with the tetraspanin CD9. Cells were transfected with EGFP-CEP55, stained with an Alexa-fluor568-coupled antibody against CD9, and analyzed by fluorescence microcopy 16 h after transfection. Shown is one representative image.

## Slide 4
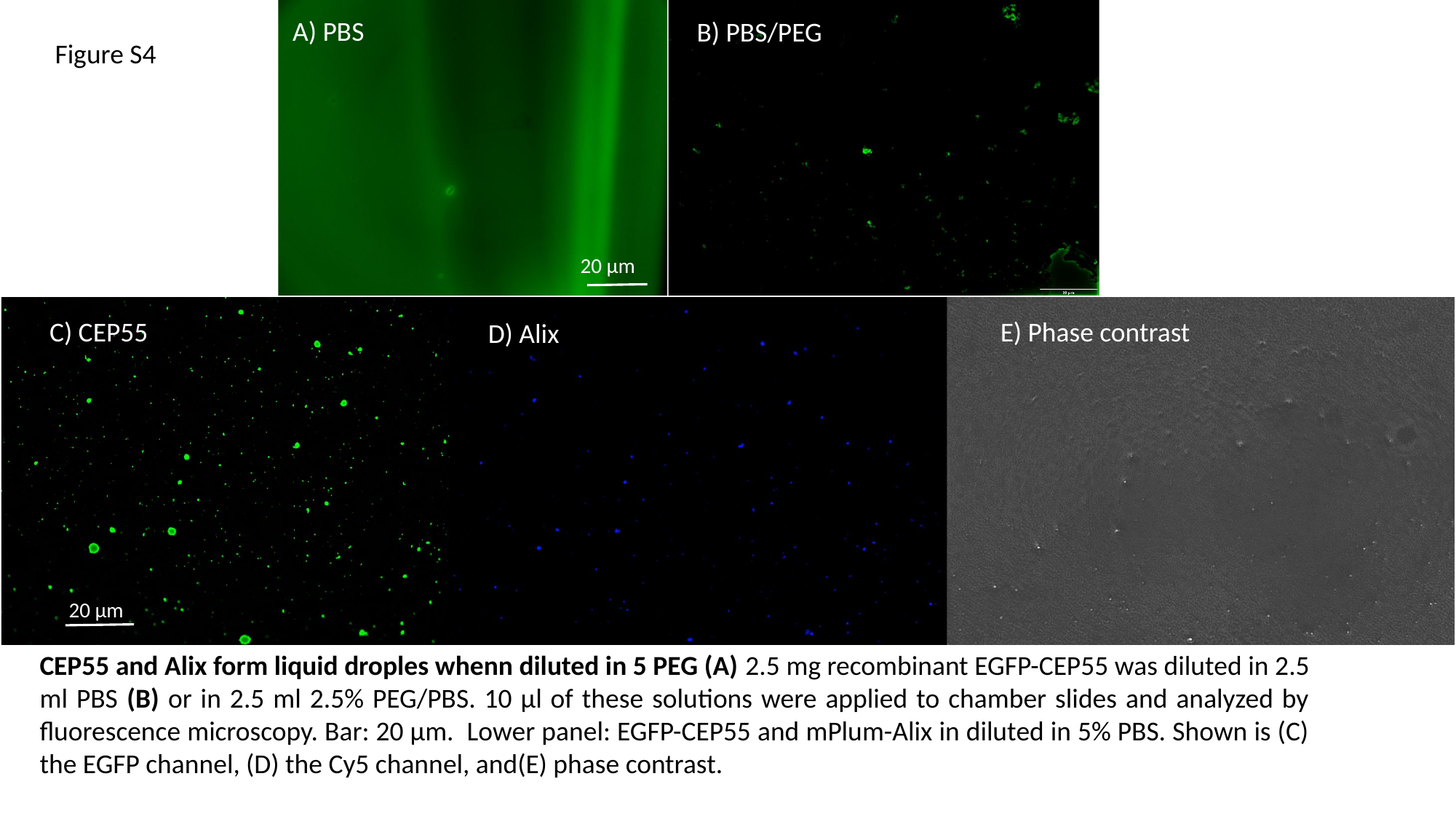

A) PBS
B) PBS/PEG
Figure S4
20 µm
C) CEP55
E) Phase contrast
D) Alix
20 µm
CEP55 and Alix form liquid droples whenn diluted in 5 PEG (A) 2.5 mg recombinant EGFP-CEP55 was diluted in 2.5 ml PBS (B) or in 2.5 ml 2.5% PEG/PBS. 10 µl of these solutions were applied to chamber slides and analyzed by fluorescence microscopy. Bar: 20 µm. Lower panel: EGFP-CEP55 and mPlum-Alix in diluted in 5% PBS. Shown is (C) the EGFP channel, (D) the Cy5 channel, and(E) phase contrast.

## Slide 5
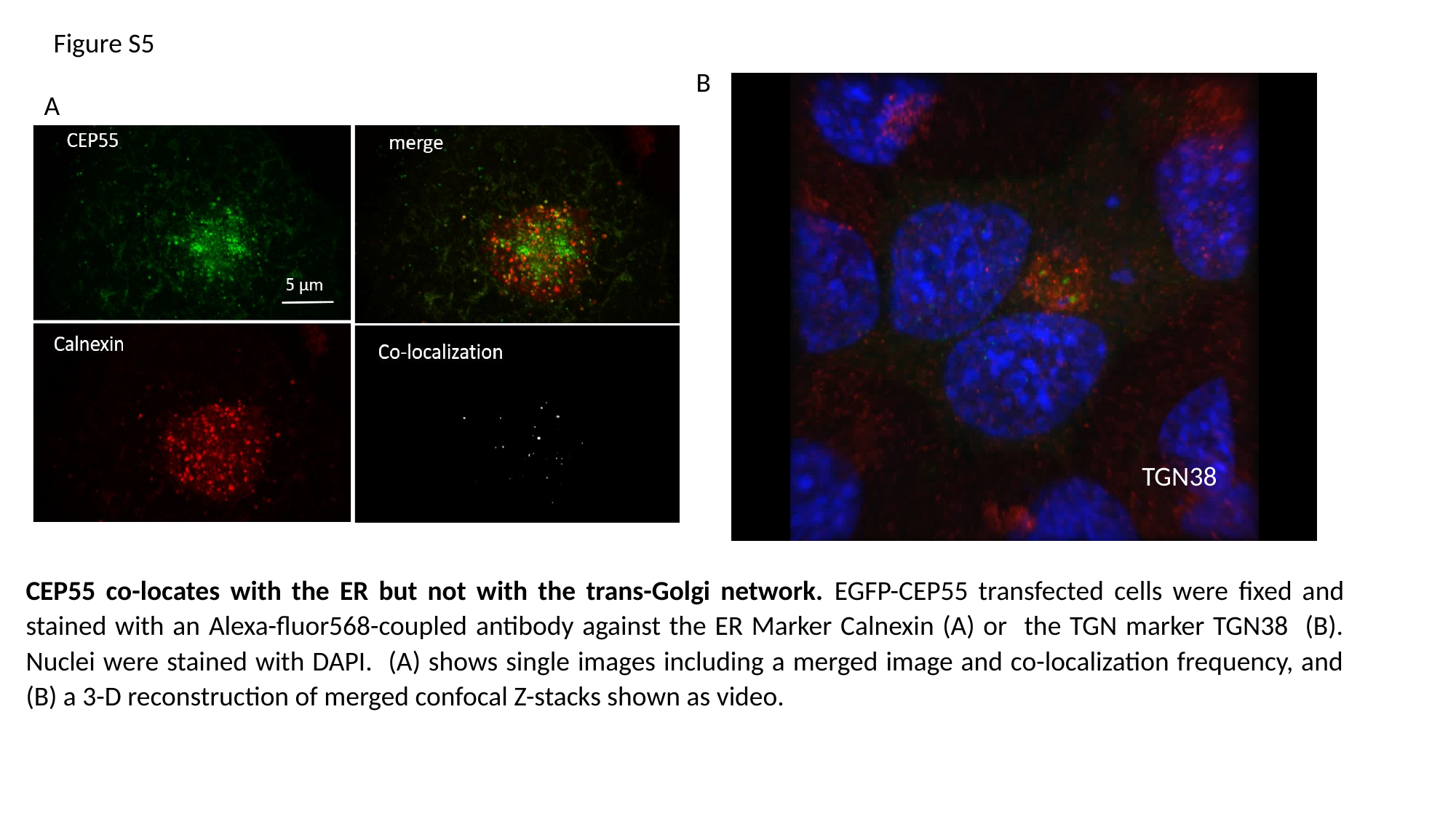

Figure S5
B
A
TGN
TGN38
CEP55 co-locates with the ER but not with the trans-Golgi network. EGFP-CEP55 transfected cells were fixed and stained with an Alexa-fluor568-coupled antibody against the ER Marker Calnexin (A) or the TGN marker TGN38 (B). Nuclei were stained with DAPI. (A) shows single images including a merged image and co-localization frequency, and (B) a 3-D reconstruction of merged confocal Z-stacks shown as video.

## Slide 6
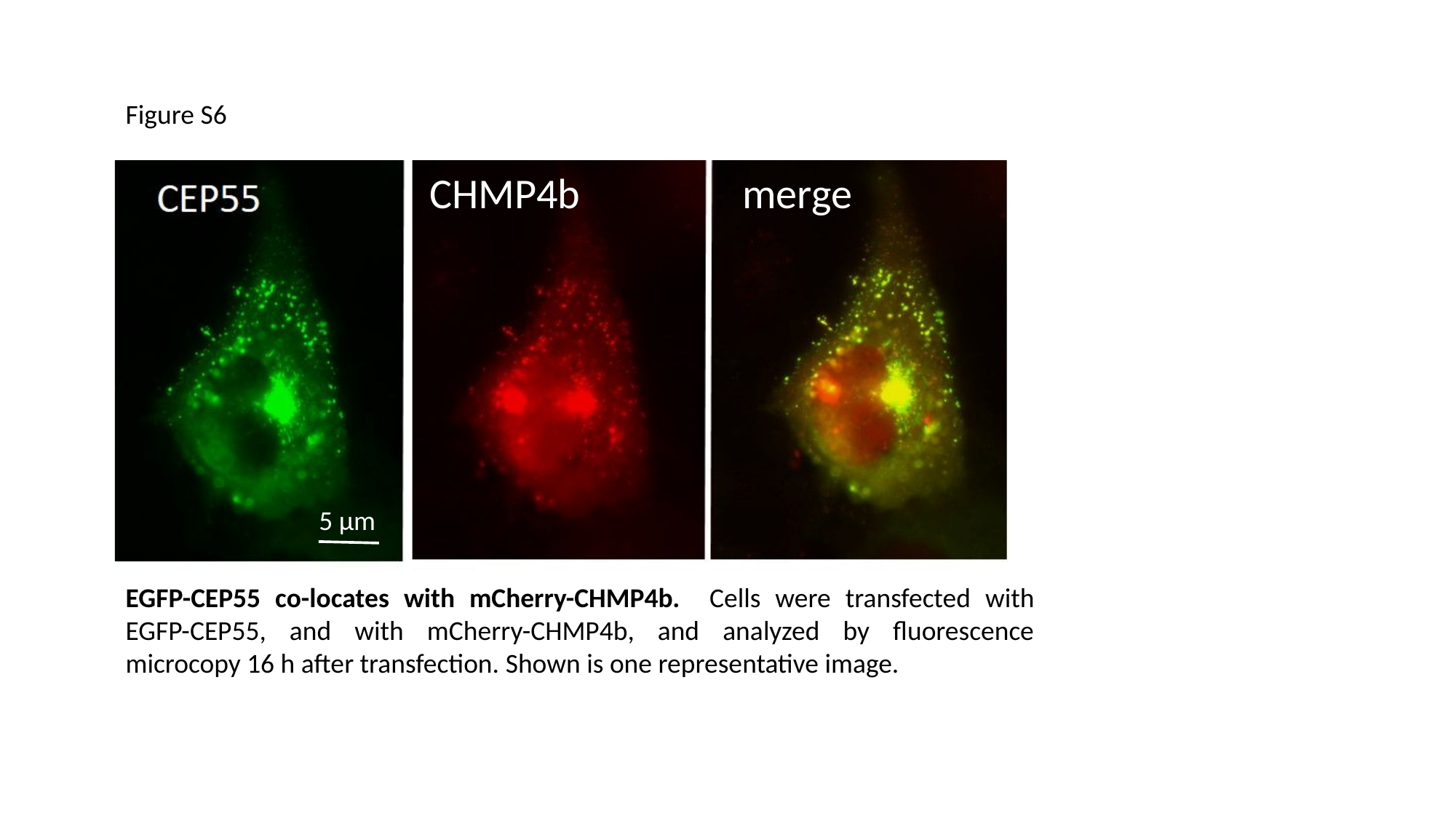

Figure S6
CHMP4b
merge
5 µm
EGFP-CEP55 co-locates with mCherry-CHMP4b. Cells were transfected with EGFP-CEP55, and with mCherry-CHMP4b, and analyzed by fluorescence microcopy 16 h after transfection. Shown is one representative image.

## Slide 7
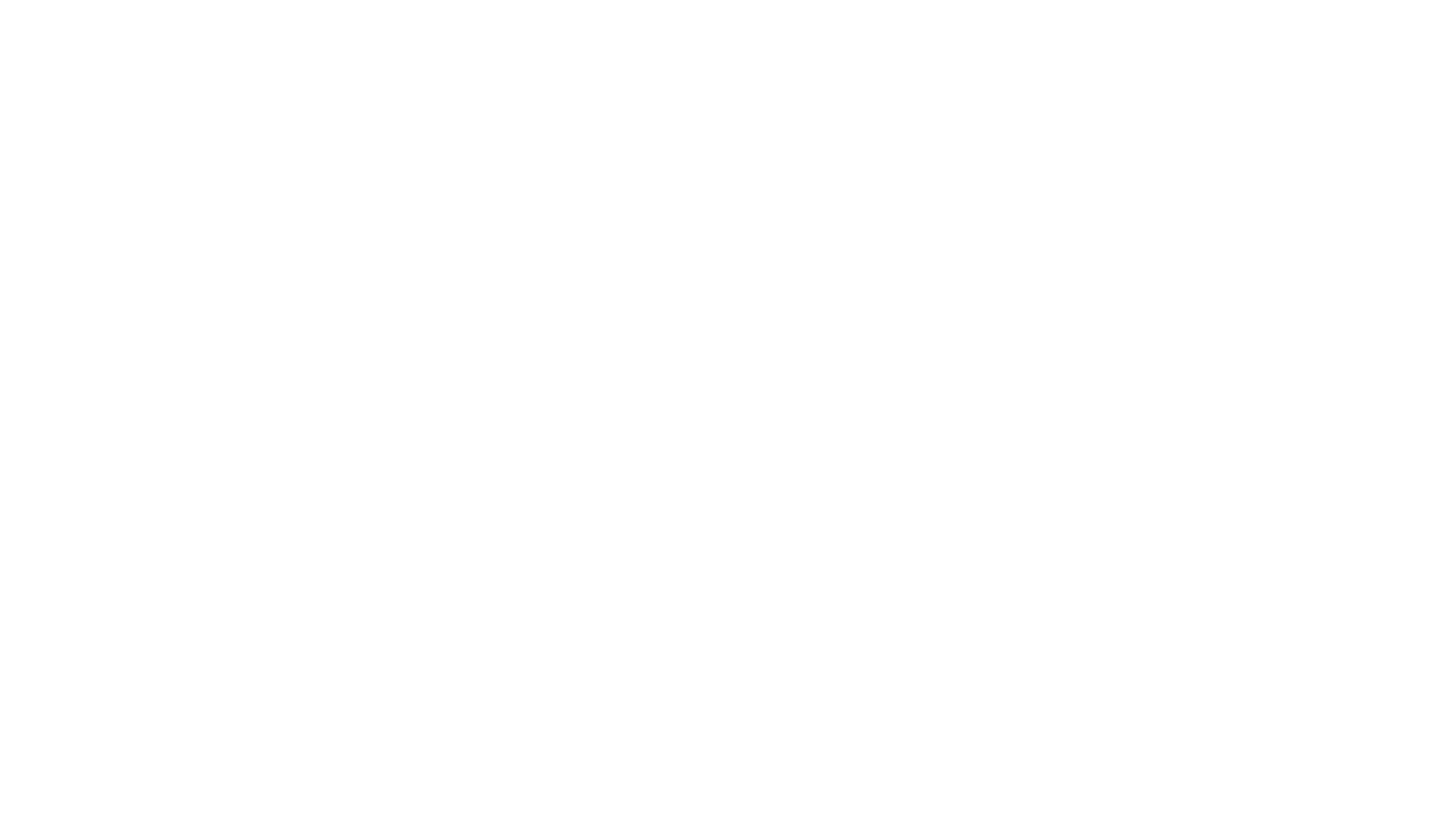

Supplement: Supplementary file 1 — Supporting Information [file JEV2-14-e70046-s001.pptx]
